# Auxin does not affect a suite of morphological or behavioral phenotypes in two wild-type *C. elegans* strains

**DOI:** 10.17912/micropub.biology.000307

**Published:** 2020-10-08

**Authors:** Troy A McDiarmid, Lexis D Kepler, Catharine H Rankin

**Affiliations:** 1 Djavad Mowafaghian Centre for Brain Health, University of British Columbia, 2211 Wesbrook Mall, Vancouver, British Columbia V6T 2B5, Canada; 2 Department of Psychology, University of British Columbia, 2136 West Mall, Vancouver, British Columbia V6T 1Z4, Canada

**Figure 1. Lifelong Auxin exposure does not affect a suite of phenotypes scored by the Multi-Worm Tracker in N2 and PD1074 wild-type C. elegans strains:**
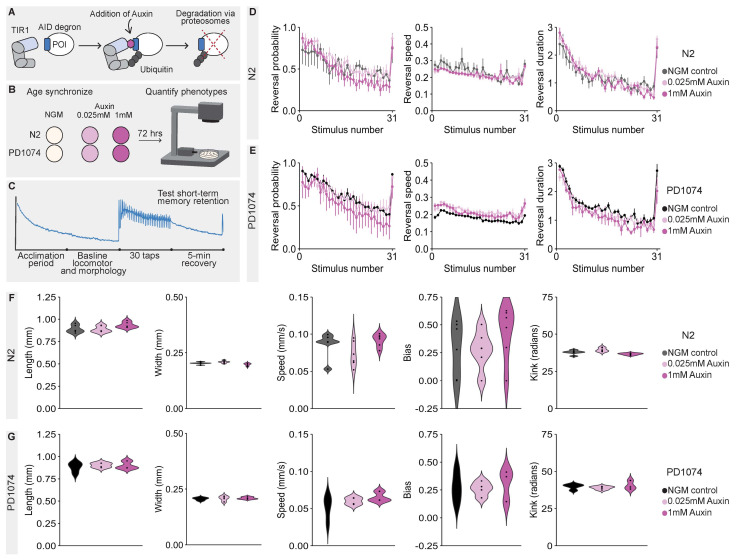
The Auxin-Inducible Degradation (AID) system relies on genetically tagging a protein of interest (POI) with an AID degron peptide tag and transgenic expression of the inducible E3 ubiquitin ligase TIR1 (A). The presence of Auxin triggers TIR1 to polyubiquitinate the POI, causing subsequent protein degradation (A). N2 and PD1074 animals were age synchronized onto standard Nematode Growth Media (NGM) plates or 0.025mM or 1mM Auxin plates (B). Animals were subjected to a short-term habituation behavioral paradigm and (C) the Multi-Worm Tracker (MWT) was used to quantify the effects of Auxin on morphological and behavioral phenotypes. For N2 and PD1074 animals, Auxin exposure at either 0.025mM or 1mM did not affect initial response sensitivity or habituation of response probability, speed, or duration (D and E), or 5 commonly studied morphological (animal length and width) and locomotion (movement speed, directional bias, and degree of kink in body posture (related to curvature of body posture)) phenotypes (F and G). A detailed description of all phenotypes can be found in the MWT user guide (Swierczek *et al.* 2011).

## Description

The Auxin-Inducible Degradation (AID) system is a powerful technique in the *C. elegans* toolkit that enables conditional and reversible protein depletion with high temporal and spatial specificity (Zhang *et al.* 2015; Martinez *et al.* 2020; Ashley *et al.* 2020; Martinez and Matus 2020). This system relies on tagging a gene of interest with a short AID degron sequence and transgenic expression of TIR1, an inducible E3 ubiquitin ligase normally found only in plants (Nishimura *et al.* 2009; Zhang *et al.* 2015). Upon exposure to the plant-derived hormone Auxin, TIR1 is activated and targets AID-tagged proteins for proteasomal degradation (Nishimura *et al.* 2009; Zhang *et al.* 2015) (Figure 1A). While there are qualitative reports that Auxin does not overtly affect the morphology or behavior of wild-type *C. elegans* (Zhang *et al.* 2015), this has not been quantitatively assessed. Determining whether Auxin significantly affects *C. elegans* morphology and behavior, even in subtle ways, is important given the *C. elegans* community’s rapid uptake of the AID system (Kasimatis *et al.* 2018; Nance and Frøkjær-Jensen 2019; Ashley *et al.* 2020; McDiarmid *et al.* 2020).Here, we use our high-throughput machine vision tracking system, the Multi-Worm Tracker (MWT) (Swierczek *et al.* 2011), to investigate whether exposure to Auxin affects a suite of morphological, locomotor, mechanosensory, and short-term habituation learning phenotypes in our lab’s derivative of Bristol N2 wild-type worms and the CGC wild-type reference strain, PD1074 (Yoshimura *et al.* 2019) (Figure 1B).

We report that lifelong exposure to 0.025mM or 1mM Auxin does not significantly affect any of the objectively quantified phenotypic features in either N2 or PD1074 wild-type strains (Figure 1D-G). We tested 1mM Auxin as it is commonly used to quickly and fully degrade AID tagged proteins, whereas, in our experience, 0.025mM Auxin still enables protein degradation but is dilute enough to allow AID tagged proteins to be re-expressed within 24 hours (hrs) of the animals being off Auxin. The Auxin plates used were indeed effective, as plates from the same batch were able to induce lethality in the CA1210 (*dhc-1::AID::GFP*) positive control strain (this is expected due to ubiquitous degradation of the essential dynein heavy chain DHC-1) (Zhang *et al.* 2015). An additional concern has been that Auxin is dissolved in ethanol (Martinez *et al.* 2020), and, as many of us know first-hand, ethanol can indeed alter animal behavior (Mitchell *et al.* 2007; Spear 2018). Importantly, we find that the ethanol used to dissolve Auxin does not affect these phenotypes when tested on agar plates, likely because the ethanol evaporates during the 72 hrs plates are allowed to dry. NOTE: The ethanol that Auxin is dissolved in will likely affect these phenotypes if using liquid culture (not tested here).

We hope this article will serve as a resource to show that with proper preparation, Auxin can be used with the AID system for effective protein depletion in *C. elegans* with minimal effects on a suite of morphological and behavioral phenotypes studied by the community. However, we still recommend the inclusion of a wild-type on Auxin negative control for behavioral experiments to ensure there is nothing wrong with the Auxin solution that may affect behavior (e.g. contamination).

## Methods

Auxin administration

Auxin treatment was performed by transferring animals to bacteria-seeded NGM plates containing Auxin. A 400 mM stock solution of Auxin indole-3- acetic acid (IAA) (Thermo Fisher, Alfa Aesar™ #A1055614) was created by dissolving Auxin in ethanol. The Auxin stock was then diluted into separate flasks of molten NGM agar to final concentrations of 0.025mM and 1mM (molten NGM was cooled to ~50°C before the addition of Auxin), and were poured into Petri plates. Auxin plates were allowed 72 hrs to dry in the dark, and were then seeded with 50 µl of OP50 liquid culture 48 hrs before use. Animals were continuously exposed to Auxin as they were age synchronized (as described below) onto Auxin plates and tested at 72 hrs post-lay.

Behavioral assays

Animals were age synchronized onto NGM and Auxin Petri plates which were seeded with 50 µl of OP50 liquid culture and dried in the dark for 72 hrs. Four to 6 plates were synchronized for each condition by picking five gravid adults onto each plate and allowing the animals to lay eggs for 4 hrs before removal (resulting in 50-100 worms per plate) (Figure 1B). Animals were then maintained in the dark in a 20°C incubator for 72 hrs.

The behavioral paradigm (Figure 1C) consisted of a 5-minute (min) acclimation period followed by a 5-min period to assess morphological and baseline locomotion phenotypes. Thirty mechanosensory stimuli were then administered to the side of the Petri plate by the MWT’s automated piston at a 10 second (sec) interval. In response to the mechanosensory stimuli, animals respond by briefly crawling backwards then resuming forward locomotion (i.e. a reversal response) (Rankin *et al.* 1990), allowing multiple features of response sensitivity and habituation to be assessed (Swierczek *et al.* 2011; McDiarmid *et al.* 2020). Following the 30th stimulus, animals were allowed to recover for 5 mins. A final stimulus was then administered to test spontaneous recovery from short-term habituation (short-term memory retention).

Multi-Worm Tracker behavioral analysis and statistics

We used the MWT software (version 1.2.0.2) for stimulus delivery and image acquisition (Swierczek *et al.* 2011), and Choreography software (version 1.3.0_r103552) for phenotypic quantification. The filters, “–shadowless”, “–minimum-move-body 2”, and “–minimum-time 20” were used to identify animals that moved ≥ 2 body lengths and were tracked for ≥ 20 secs. Morphology and baseline locomotion features were identified using standard choreography output commands (Swierczek *et al.* 2011). Reversals that occurred within 1 sec after the administration of a mechanosensory stimulus were identified using the MeasureReversal plugin (Swierczek *et al.* 2011). Comparisons of “final response” comprised the average of the final three stimuli. For a complete description of the 26 phenotypic features, see the MWT user guide (https://sourceforge.net/projects/mwt/). All data is freely available upon request. Custom R scripts organized and summarized Choreography output files which are freely available at https://github.com/troymcdiarmid/MWT_Wildtype_Auxin/releases/tag/v1.0. (An archived version of these data and scripts are available on Caltech Data https://doi.org/10.22002/d1.1639). Condition blinding was not necessary as the MWT scores behavior objectively (Swierczek *et al.* 2011). Phenotypic features were pooled across the 4-6 plate replicates (50-100 animals per plate) for each strain and means of each condition were compared with an unpaired t-test and Benjamini-Hochberg control of the false discovery rate at 0.01. Final figures were generated using the ggplot2 package in R (Wickham 2016). Images taken by the MWT were used to confirm lethality of CA1210 (*dhc-1::AID::GFP*) animals after 72 hrs of Auxin exposure.

## Reagents

N2 wild-type, PD1074 wild-type, and CA1210 *dhc-1*(*ie28*[*dhc-1*::degron::GFP]) I; *ieSi57* II. All strains are available from the CGC.
